# Interhemispheric transcallosal intervenous approach to a pineal region tumor

**DOI:** 10.3171/2021.4.FOCVID2120

**Published:** 2021-07-01

**Authors:** Daniel A. Donoho, Guillermo Aldave

**Affiliations:** 1 Division of Neurosurgery, Department of Surgery, Texas Children’s Hospital, Houston; and; 2 Department of Neurosurgery, Baylor College of Medicine, Houston, Texas

**Keywords:** pineoblastoma, transcallosal intervenous approach, pineal tumor

## Abstract

Pineal region tumors represent a formidable challenge to the neurosurgeon. Choosing the right approach is key to optimizing the extent of resection and minimizing surgical morbidity. In this video, the authors show an interhemispheric transcallosal approach to a pineal region tumor in a 15-year-old boy. The advantage of this corridor over posterior approaches is that it provides a nice view of the tumor plane with the venous complex, especially while dissecting tumor from the anterior aspect of the internal cerebral veins on their vertical path. Thus, this approach represents a safe and effective alternative for selected pineal tumors.

The video can be found here: https://stream.cadmore.media/r10.3171/2021.4.FOCVID2120.

## Transcript

In this video, we present an interhemispheric transcallosal intervenous approach to a pineal region tumor.

### 0:26 Case Presentation

This is a case of a 15-year-old boy with a history of headaches and double vision for a few weeks without significant past medical history (PMH). The exam did highlight an abduction deficit in the right eye and bilateral papilledema. He is otherwise intact.

The initial MRI showed a pineal region tumor causing obstructive hydrocephalus. The tumor has significant diffusion restriction and homogenous contrast enhancement. There were no other lesions in the brain or spine.

Tumor markers AFP and HCG in blood were within normal limits. The main differential diagnosis in this situation remains germinoma versus pinealoblastoma. At this point, we decided to perform an endoscopic third ventriculostomy first to address the hydrocephalus, including an endoscopic biopsy with flexible endoscope through a single right frontal burr hole.

The pathologic diagnosis came back as pineoblastoma, so in this context tumor resection is indicated as the next step in treatment, followed by craniospinal radiation and chemotherapy. Besides, in patients older than 5 years with focal disease, gross-total/near-total resection can improve overall survival and should be the goal.^1^

### 1:33 Surgical Approach Discussion

Among the options to approach this tumor, we always consider these three approaches for the pineal region: supracerebellar, occipital transtentorial, and the transcallosal intervenous approach. All these three options were considered valid and feasible for this tumor.

However, we opted for the interhemispheric transcallosal and intervenous approach, as it provides a nice view of the plane of the tumor anterior to the ICVs;^2–5^ that is usually where the tumor is going to be attached and where optimizing the view is always worth considering. Thus, this approach would allow us to have a nice view in front of the venous complex, preserving the vermian vein, the central mesencephalic vein, and of course both Rosenthal veins.

### 2:13 Patient Positioning and Incision

The patient is positioned supine with the head turned rightward parallel to the floor, so the right hemisphere will fall down opening the interhemispheric corridor naturally.

### 2:24 Microsurgical Approach and Dissection of the Corridor

Under the microscope, the right side is on the bottom of the screen; thus, anterior is on the right upper corner of the screen and posterior is left lower corner.

The dissection of the interhemispheric fissure places us into the corpus callosum. Neuronavigation is very helpful to drive the callosotomy exactly where we had defined it preoperatively, right above the splenium. This places us into the left lateral ventricle apparently. To confirm this, especially before the dissection of the velum interpositum in the midline, we expose also the right lateral ventricle as we can see.

Once we have both lateral ventricles exposed, we move forward with the exposure of the tela choroidea in the roof of the third ventricle. Doing the midline dissection close to the splenium of the corpus callosum it is always safe, as the body of the fornices should have already left the midline and turned into the crus of the fornix in the anterior wall of the atrium.^6^

In this dissection of the velum interpositum, we can identify all the layers of the tela choroidea. The opening of the upper sheath exposes the vascular layer with the medial posterior choroidal artery that at this point has already given branches to the thalamus and is basically supplying the choroid plexus. In this layer, we will also find both internal cerebral veins. We always take time to dissect the tela and the arachnoid as at the beginning it may be hard to distinguish the arachnoid from the veins themselves.^6^ Once both ICVs come together in midline is a good anatomical reference to know when the body of the fornix leaves midline and turn into the crus. For bigger tumors where a larger corridor may be desirable, opening the rest of the tela anteriorly and the interforniceal fissure is also a good option.

### 4:27 Tumor Resection

Finally, once we open the lower sheath of the tela, we expose the tumor. The tumor is brown, moderately vascular alternating areas that are soft, with others more fibrous, as expected.

The initial debulking with suction is very efficient and allows us to expose the anterior two-thirds of the third ventricle. Besides, we also notice a white structure that is crossing midline that should correspond to the superior commissure that surrounds the pineal region, the habenular commissure. The posterior commissure is seen at the bottom right over the tectal plate.

This is a nice view that shows both commissures that we find around the pineal region, the superior commissure, or habenular commissure, and the posterior commissure in the inferior margin of the pineal region, on top of the tectal plate.^6^ The habenular commissure is hard to identify in pineal tumors as it is most of the time embedded into the tumor, but this tumor is relatively small and has kept it and pushed it superiorly.

As we move forward with the dissection and resection following the tectal plate, we expose the aqueduct, having a nice view that confirms the resection of the component of the tumor that was blocking it. Having done the dissection of the tectal plate and exposing the aqueduct, we cover and protect it with a cottonoid patty for the rest of the case.

We continue with the inspection and dissection on both sides of the field where the tumor is attached to the walls of the third. On the margins or periphery of the tumor, this turns more fibrous, as expected. The dissection with suction is not as effective as it was with the soft component, but we can still get a nice plane on both sides with patience and gentle dissection.

Finally, we leave the last piece to the end since it was attached to the left internal cerebral vein as seen on the preoperative MRI. We can see the big blue structure in the left side of the surgical field. In our experience, even small tumors like this one can be really attached to the walls of the vein.

This section of the video demonstrates the added value of the interhemispheric transcallosal approach for this particular tumor dissection. We are trying to dissect the tumor from the anterior and inferior side of the left internal cerebral vein; this side would be hard to expose coming from a posterior approach, especially in the vertical trajectory of the vein where the tumor is attached.

We dissect the tumor as much as we can until we lose the nice plane between the tumor and the vein. We insist to make sure there is no good plane that can be dissected, so we decide to trim it or shave the last piece. While constantly maintaining a nice view of the vein as our reference, we continue with the coagulation of the tumor, trimming it with microscissors until we reach the plane with the wall of the vein.

In this part, we take time to make sure we have good control all around it before we cut it out, taking advantage of the nice view that this approach provides for this particular case. Finally, we coagulate the implantation of the tumor.

### 8:25 Final Hemostasis, Inspection, and Closure

Thus, we get a reasonable gross-total resection of the tumor. We inspect the surgical field all around it; we see the right lateral wall with the right ICV, the left lateral wall with left ICV, and choroid plexus on the roof of the third ventricle. This is the final view of the surgical cavity with both ICVs.

### 8:55 Postoperative Course and Management

The postoperative MRI showed a complete resection of the tumor without complications. The patient recovered very well. He went home within 5 days after the surgery without additional deficits except for an expected Parinaud syndrome. Pathology confirmed the diagnosis of pineoblastoma. He completed treatment with craniospinal radiation, 23-Gy and 30-Gy boost in the surgical field, followed by chemotherapy with autologous bone marrow transplant. Currently, his Parinaud syndrome has resolved and there is no evidence of tumor recurrence.

## Author Contributions

Primary surgeon: Aldave. Assistant surgeon: Donoho. Editing and drafting the video and abstract: both authors. Critically revising the work: both authors. Reviewed submitted version of the work: Aldave. Approved the final version of the work on behalf of both authors: Aldave. Supervision: Aldave.
